# Comparison between the brief seven-item and full eating disorder examination-questionnaire (EDE-Q) in clinical and non-clinical female Norwegian samples

**DOI:** 10.1186/s40337-023-00920-x

**Published:** 2023-11-02

**Authors:** Lasse Bang, Morten Nordmo, Magnus Nordmo, Karianne Vrabel, Marit Danielsen, Øyvind Rø

**Affiliations:** 1 Department of Child Health and Development, Norwegian Institute of Public Health, Oslo, Norway; 2 Regional Department for Eating Disorders, Division of Mental Health and Addiction, Oslo University Hospital, Oslo, Norway; 3 Department of Leadership and Organisational Behaviour, Norwegian Business School (BI), Oslo, Norway; 4 Department of Educational Science, University of South-Eastern Norway, Notodden, Norway; 5 Department of Psychology, University of Oslo, Oslo, Norway; 6 Modum Bad Psychiatric Hospital, Vikersund, Norway; 7 Eating Disorder Unit, Department of Psychiatry, Levanger Hospital, Hospital Trust Nord-Trøndelag, Levanger, Norway; 8 Division of Mental Health and Addiction, Institute of Clinical Medicine, University of Oslo, Oslo, Norway

**Keywords:** Eating disorders, Eating disorder examination questionnaire, Assessment, Psychometric

## Abstract

**Background:**

The Eating Disorder Examination-Questionnaire (EDE-Q) is among the most widely used self-report measures of eating disorder (ED) psychopathology. There is a need for brief versions of the EDE-Q that can be used for general assessment and screening purposes. A three-factor 7-item version (EDE-Q7) seems particularly promising but there is a need for more well-powered studies to establish the psychometric properties in both patient and community samples. Moreover, comparing the EDE-Q7 with the full EDE-Q would be beneficial in determining its utility. In the present study, we provide a psychometric comparison between the brief EDE-Q7 and the full EDE-Q in a large sample of both patients and community comparisons.

**Methods:**

We pooled available datasets collected in Norway to amass a large female sample comprising both patients (*n* = 1954, *M*_age_ = 28 years) and community comparisons (*n* = 2430, *M*_age_ = 31 years). We investigated the psychometric properties of both versions, including their internal consistency, factor structure, and ability to discriminate between patients and community comparisons.

**Results:**

The EDE-Q7 showed similar distributions of scores compared to the full EDE-Q but produced higher scores. Results indicated that the EDE-Q7 have acceptable internal consistency and is adequately able to discriminate between clinical and non-clinical samples. A cut-off threshold of 3.64 was optimal in discriminating between patients and comparisons. We also found support for the three-factor solution for the EDE-Q7, indicating good structural validity. In contrast, we did not find support for the originally proposed four-factor solution of the full EDE-Q.

**Conclusions:**

We find that the brief EDE-Q7 performs close to the full EDE-Q in several respects. Our findings indicate that the brief EDE-Q7 may be a viable alternative to the full EDE-Q in situations where response burden is an issue (e.g., epidemiological studies). However, the EDE-Q7 may hold limited value over the full EDE-Q in clinical settings, due to the small number of items and lack of assessment of behavioral features.

**Supplementary Information:**

The online version contains supplementary material available at 10.1186/s40337-023-00920-x.

## Abstract (plain language summary)

The Eating Disorder Examination-Questionnaire (EDE-Q) is a widely used self-report measure to assess eating disorder symptoms in clinical and research settings. One limitation of the EDE-Q is its length (28 questions), which can preclude its use in research studies where assessments need to be short. A brief seven-question version has been proposed (referred to as the EDE-Q7), but few studies have evaluated its usefulness in comparison to the full EDE-Q. In our study we aimed to provide a comparison between the brief EDE-Q7 and the full EDE-Q among female Norwegian patients and non-patients. The brief EDE-Q7 produced similar responses compared to the full EDE-Q and performed well in tests of its integrity as a measure. Our findings suggest that the EDE-Q7 may serve as a viable alternative to the full EDE-Q for brief assessment and screening purposes.

**Supplementary Information:**

The online version contains supplementary material available at 10.1186/s40337-023-00920-x.

## Background

The Eating Disorder Examination-Questionnaire (EDE-Q) [1] is among the most widely used self-report measures of eating disorder (ED) psychopathology. The EDE-Q consists of 28 items assessing core attitudinal features and behaviors of EDs. It consists of four subscales: restraint, eating concern, weight concern, and shape concern, which are averaged to produce a global score. The EDE-Q is extensively used as a general assessment of ED psychopathology in both clinical and non-clinical populations.

Studies have demonstrated sound psychometric properties of the EDE-Q, including satisfactory reliability and validity [2–5]. However, studies have consistently failed to replicate the originally proposed four-factor structure of the EDE-Q [6]. Instead, studies have found support for a variety of factor solutions, with a three-factor solution being the most commonly supported [3, 6–15].

A major limitation of the EDE-Q is its length, which often precludes its use in research settings where response burden is a concern. As a result, assessments of EDs are often omitted due to the lack of brief assessment measures. Alternatively, EDs are assessed using study-specific items which hampers comparisons across studies. Consequently, there is a need for brief versions of the EDE-Q that can be used for general assessment and screening purposes.

Several shortened versions of the EDE-Q have been proposed in the literature [6, 8, 11, 16–19]. The extent to which these have been utilized and tested vary considerably. Among the proposed versions, some are particularly promising for brief assessment and screening purposes.

The EDE-Q7 [11] is composed of seven items derived from the original EDE-Q, retaining the same response scale (0–6) referencing the past 28 days. These items yield three distinct factors (subscales) which differ from the original EDE-Q structure. Its brevity makes it particularly relevant for general epidemiological assessment and screening purposes. Of note, the behavioral items of the original EDE-Q (e.g., binge-eating and purging) are not included. This limits its use in contexts where the assessment of such behaviors is important (e.g., clinical work). The EDE-Q7 has been tested across a range of samples, encompassing both patient and community groups. These studies have supported its psychometric soundness [3, 10–12, 14, 20–31].

Similarly, the EDE-Q8 [17] consists of eight items derived from the original EDE-Q. Like the EDE-Q7, it retains the original response scale. However, the EDE-Q8 was designed to align with the original four-factor structure of the full EDE-Q. Like the EDE-Q7, behavioral items are not included. Additionally, it has been adapted for use with children [32]. Several studies have supported its psychometric soundness [17, 20, 32].

The 12-item EDE-Q short form (EDE-QS) represents an adaptation of the original EDE-Q into a brief format suitable for routine outcome assessment [16]. The response scales have been transformed into a four-point scale (0–3) referencing the past seven days. This change may be particularly beneficial in contexts where frequent repeated assessments are required. Authors of the EDE-QS have suggested a five-factor solution, and none of the factors replicate the original EDE-Q subscales. The EDE-QS retains some of the behavioral items, allowing it to assess for ED-related behaviors as well. Studies have provided support for the psychometric properties of the EDE-QS [16, 33–35].

Lastly, the EDE-Q13 [18] is essentially the EDE-Q7 version with the addition of the original EDE-Q behavioral items. It generates five factors, with the two additional factors (compared to the EDE-Q7) reflecting binge-eating and purging behaviors. Two studies have supported its psychometric soundness [18, 36], but further research is required to substantiate its psychometric properties. We note that several other abbreviated EDE-Q versions have been suggested in the literature (e.g., [6, 8, 19]).

Each of the brief EDE-Q version may have its own unique merits. For instance, the EDE-Q7 and EDE-Q8 may be particularly suited for epidemiological research, where a concise tool for general assessment and screening of ED psychopathology is essential. These versions also maintain the same response scale format as the original EDE-Q, which may be beneficial in certain situations. However, their brevity and omission of behavioral items limit their utility in clinical settings. The EDE-QS may also offer value in epidemiological contexts. Its inclusion of behavioral items and focus on the past seven days renders it particularly useful for routine outcome assessment in clinical settings. Although initial studies indicate that the EDE-Q7 [20], EDE-Q8 [20], and EDE-QS [35] can effectively differentiate between patients and non-patients, further research is required to ascertain their screening accuracy.

Although all brief versions of the EDE-Q have undergone evaluation in various studies, the EDE-Q7 stands out for its well-documented psychometric properties across a range of samples [3, 10–12, 14, 20–31]. The EDE-Q7 is also the most extensively utilized and studied tool in this context. Furthermore, some direct comparisons have demonstrated the superior performance of the EDE-Q7 when compared to certain other brief versions of the EDE-Q [20, 24, 25]. Being the shortest of all aforementioned brief EDE-Q versions, The EDE-Q7 may offer particular benefit for epidemiological studies that require swift and concise general assessment and screening for ED psychopathology.

There is a need for more well-powered studies to establish the psychometric properties of the EDE-Q7 in both clinical and non-clinical populations. The ability of the EDE-Q7 to distinguish between clinical and non-clinical samples also needs to be explored, as this will elucidate its suitability as a screening tool. Furthermore, comparisons between the EDE-Q7 and the full EDE-Q would be helpful in determining the relative benefits of each one.

In the present study, our aim was to provide a comparison between the brief EDE-Q7 and the full EDE-Q. We pooled several available datasets collected in Norway to amass a large female sample comprising both patients and community comparisons. We investigated the psychometric properties of both versions, including their internal consistency, factor structure, and ability to discriminate between patients and community comparisons. The overall goal was to evaluate the suitability of the EDE-Q7 as a brief general measure of ED psychopathology.

## Methods

### Datasets

We pooled existing datasets where EDE-Q data from Norwegian patient and community samples (all females) were available. Data on patients admitted to six ED treatment units in Norway during the period 2011–2020 were extracted from local electronic patient databases. These units provide out- or inpatient specialized treatment to patients (children and adults) with severe EDs. The units are geographically spread throughout Norway and represent most specialized treatment units for EDs in Norway. These data were originally collected during admission to treatment, typically within the first two weeks of admission. All patients in this sample had a clinically defined ED diagnosis according to ICD-10 criteria. Subsamples of these patient data have been included in prior studies (e.g., 5).

Data on community comparisons (henceforth referred to as “comparisons”) were extracted from five separate Norwegian research studies conducted in the period 2008–2020. Recruitment of these comparisons varied between studies. One study [37] approached a national representative community sample via a random selection by the Norwegian Population Register and included 37% of invitees. Two studies [38, 39] recruited participants nationally via online platforms (e.g., Facebook) and locally with flyers at universities. The two remaining studies approached and recruited students from local universities, colleges or high schools [40, 41]. With the exception of the study by Friborg and colleagues [37]—which also constitute the largest community sample—the samples should be regarded as convenience samples, and are unlikely to be representative of the Norwegian population. Our data should therefore not be used to determine norms for the EDE-Q and EDE-Q7; but instead provide a basis for comparisons between the measures.

In the individual studies, efforts were made to exclude patients from the comparison samples (e.g., with the question “do you currently have an ED?”, or by excluding individuals who met case criteria). However, because patients and comparisons were recruited separately there is a risk of overlap between samples (e.g., an admitted patient was included as a comparison in one of the studies at another point in time), although we estimate this overlap to be minimal. More details on the individual studies and their samples are found in the original publications [37–41].

We extracted raw EDE-Q scores (all 28 items), age, sex and body mass index (BMI; kg/m^2^) for all participants. For patients, we also extracted diagnostic information and treatment level (inpatient vs. outpatient) where this information was available.

### Participants

A total of 1954 participants were included in the patient sample, while 2430 were included in the comparison sample. For 37% of the patient sample, we had no information regarding their specific ED diagnosis, due to incomplete records (i.e., diagnostic information was never registered in the electronic patient record). Of all participants in the patient sample for which we had diagnostic information according to ICD-10, 552 (45%) were diagnosed with anorexia nervosa (AN; typical or atypical), 444 (36%) were diagnosed with bulimia nervosa (BN; typical or atypical), and 234 (19%) were diagnosed with other EDs—including eating disorder not otherwise specified. Most patients (62%) were admitted to inpatient (as opposed to outpatient) treatment. We only included participants for whom valid EDE-Q global *or* subscale scores could be computed (see below).

### EDE-Q and EDE-Q7

The Norwegian EDE-Q (version 6.0) was previously developed through a translation/back-translation procedure [41], and is extensively used for research and clinical purposes in Norway. The EDE-Q assesses core attitudinal features and behaviors of EDs during the past 28 days. Except for items probing the frequency of ED-related behaviors, responses are rated on a 7-point scale ranging from 0 to 6. Originally a four-factor solution was suggested [1], and scores are averaged across these factors to produce the following subscales: restraint (items 1, 2, 3, 4 and 5), eating concern (items 7, 9, 19, 20 and 21), weight concern (items 8, 12, 22, 24, 25), and shape concern (items 6, 8, 10, 11, 23, 26, 27, 28). The restraint subscale comprises items that assess endorsement of restrictive behaviors such as dietary restriction, food avoidance, and eating avoidance. The eating concern subscale assesses concerns over eating, including preoccupation with eating, fear of losing control over eating, and guilt about eating. Finally, the shape and weight concern subscales assess concerns related to one’s body shape and weight, including preoccupation with shape or weight, dissatisfaction with shape or weight, and discomfort seeing one's body shape or weight. These subscales are averaged to compute the EDE-Q global score, which provides an overall assessment of ED psychopathology. The full English and Norwegian versions are available online (see [42, 43] for links to the full versions).

We computed an average score for each of the four subscales, requiring that valid scores be available for the majority of items within each subscale. Next, we computed the EDE-Q global score by averaging the subscale scores, requiring that valid scores were available for at least two of the subscales. A global score of > 2.5 has previously been established as an optimal cut-off to discriminate between patients and comparisons for the Norwegian EDE-Q [5]. The number of missing values for individual EDE-Q items were negligible, ranging from 0.2 to 1.9% for patients and 0.1–0.8% for comparisons (see Additional file 1: Table S1 for detailed information on missing values).

The brief EDE-Q7 is an abbreviated 7-item version of the full EDE-Q [11, 22, 44]. This version consists of three subscales: dietary restraint (items 1, 3 and 4), shape/weight overvaluation (items 22 and 23) and body dissatisfaction (items 25 and 26). We computed an average score for each of these three subscales, and a global EDE-Q7 score by averaging the three subscales. We note that prior studies have not computed an EDE-Q7 global score [22], but we wanted to determine if a global score can be meaningfully used to discriminate between patients and comparisons (i.e., for screening purposes). Because no prior studies have explored the Norwegian EDE-Q7 and there are few items in this version, we only included participants for which all 7 items were available for analyses of the EDE-Q7. We note that the datasets pooled in our study all administered the full EDE-Q. Therefore, the EDE-Q7 has not been administered in its intended form but is nested within the full EDE-Q. Administering only the 7 items in the EDE-Q may produce different scores than when a nested version is considered.

### Analyses

#### Between-group differences

Between-group differences in age, BMI, EDE-Q and EDE-Q7 scores were investigated with Welch t-tests. As distributions of scores were heavily skewed, we also performed non-parametric Mann–Whitney U tests. The magnitude of between-group differences was characterized with Cohen’s *d*. Analyses were performed in RStudio [45].

#### ROC analyses

We used receiver operator curve (ROC) analyses to determine the optimal EDE-Q and EDE-Q7 cut-off thresholds to discriminate between patients and comparisons. The ROC curves were obtained by plotting sensitivity against 1-specificity for each possible cut-off score [46]. The area under the ROC curve (AUC) was used to indicate the performance of the EDE-Q global score. A value of 0.5 on the AUC indicates discrimination no better than chance, and a value of 1.0 represents perfect discrimination. We used Youden’s statistic [47] to determine the optimal cut-off:

$${\text{max}}\left( {sensitivity + specificity} \right).$$

We investigated ROC curves for both the full EDE-Q and the brief EDE-Q7 separately, to determine the optimal cut-off thresholds for both versions. ROC analyses were performed using the pROC library [48] for RStudio [45].

#### Confirmatory factor analysis of the EDE-Q and EDE-Q7

We evaluated the factor structure of the full EDE-Q and the EDE-Q7. The multifactorial structure of the full EDE-Q was tested with a confirmatory factor analysis model based on Fairburn’s [1] original four-factor structure. The model correlated the error terms between item pairs: 7 & 8, 11 & 12, 22 & 23 as well as 27 & 28, based on their similar wording.

The factor structure of the EDE-Q7 was similarly evaluated with a confirmatory factor analysis model. The model consists of three factors [10]: dietary restraint (items 1, 3 and 4), shape/weight overvaluation (items 22 and 23), and body dissatisfaction (items 25 and 26). Preliminary analyses and modification indices revealed that the model fit improved substantially if the error terms of items 25 and 23 were allowed to covary, so we included a covariance parameter between the error terms of these items. For the EDE-Q7, we also estimated the factorial invariance, internal consistency using ρ = Raykov’s reliability coefficient *ρ* [49], average variance extracted (AVE) and inter-correlations between factors.

For all confirmatory model analyses model fit was estimated for the total sample, and for patients and comparisons separately. Model fit was assessed with a chi-square test, comparative fit index (CFI), Tucker–Lewis index (TLI), standardized root mean square residual (SRMR) and root mean square error of approximation (RMSEA). We placed special importance on RMSEA as it is sensitive to the number of free parameters [50], and estimated with maximum likelihood. We also investigated the measurement equivalence of the EDE-Q7 between patients and comparisons. We first estimated the factorial invariance for the EDE-Q7 with both exploratory factor analysis using Oblimin rotation as well as multi group confirmatory factor analysis. We proceeded to examine metric and scalar invariance if factorial invariance was established. Factor analyses were performed in Stata [51]. For all analyses, results were considered statistically significant if *p* < .05.

## Results

### Sample characteristics

Sample characteristics are presented in Table 1 along with tests of between-group differences. Age ranged from 15 to 61 years for patients and 15 to 78 for comparisons. Patients were significantly younger than the comparisons, with an average mean difference of ~ 2 years. BMI ranged from 8.86 to 63.37 for patients and 13.46 to 55.10 for comparisons, and patients had significantly lower BMI. See Additional file 1: Fig. S1 for distributions of age and EDE-Q scores across the individual study samples.

**Table 1:** Sample descriptives and between-group differences

Variable	Sample descriptives		Mean difference	Independent samples Welch t-test		
	Patients (*n* = 1954)	Comparisons (*n* = 2430)		*t* (df)	*p*	*d* (95% CI)
	*M* ± *SD*	*M* ± *SD*				
Age	28.17 ± 8.93	31.36 ± 10.42	− 3.19	− 10.37 (3778.7)	< .001	− 0.32 (− 0.39; − 0.26)
BMI (kg/m^2^)	21.49 ± 7.20	23.85 ± 4.50	− 2.36	− 11.91 (2604.4)	< .001	− 0.41 (− 0.47; − 0.35)
EDE-Q global	3.97 ± 1.31	1.25 ± 1.12	2.72	73.00 (3835.9)	< .001	2.26 (2.48; 2.48)
EDE-Q restraint	3.61 ± 1.65	1.16 ± 1.25	2.45	54.19 (3547.4)	< .001	1.70 (1.49; 1.96)
EDE-Q eating concern	3.45 ± 1.46	0.51 ± 0.87	2.94	78.20 (3016.3)	< .001	2.51 (2.76; 2.76)
EDE-Q weight concern	4.14 ± 1.56	1.53 ± 1.41	2.61	57.39 (3963.6)	< .001	1.77 (1.55; 2.04)
EDE-Q shape concern	4.71 ± 1.40	1.81 ± 1.50	2.90	65.99 (4277.1)	< .001	1.99 (1.75; 2.30)
EDE-Q7 global	4.61 ± 1.36	1.91 ± 1.51	2.70	61.71 (4210.1)	< .001	1.88 (1.65; 2.17)
EDE-Q7 dietary restraint	4.30 ± 1.92	1.73 ± 1.81	2.57	45.04 (4027.4)	< .001	1.38 (1.32; 1.45)
EDE-Q7 shape/weight overvaluation	4.69 ± 1.60	1.69 ± 1.64	3.00	60.73 (4183.6)	< .001	1.85 (1.63; 2.13)
EDE-Q7 body dissatisfaction	4.82 ± 1.56	2.30 ± 1.82	2.52	49.00 (4309.4)	< .001	1.47 (1.41; 1.54)

Results considered statistically significant if *p* < .05

*BMI* Body mass index, *d* Cohen’s *d*, *EDE-Q* Eating Disorder Examination-Questionnaire, *M* Mean, *SD* Standard deviation

### EDE-Q and EDE-Q7 scores and between-group differences

**Fig. 1:**
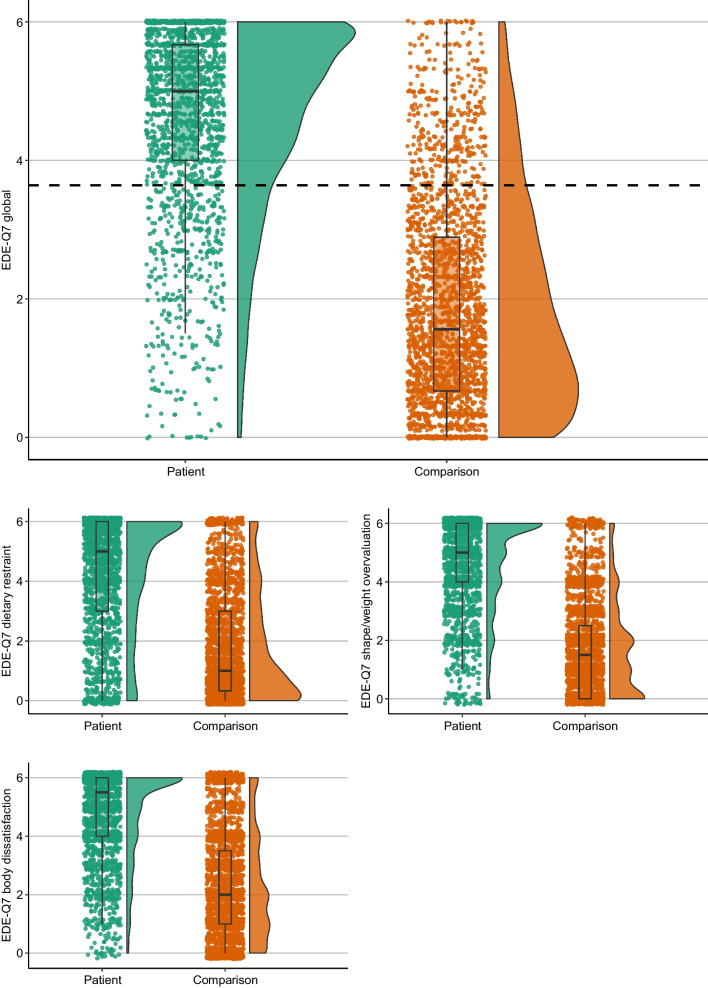
Raincloud plots showing distributions of EDE-Q7 global and subscale scores within patient and comparison groups. Dotted line signifies EDE-Q7 cut-off threshold (> 3.64)

**Fig. 2:**
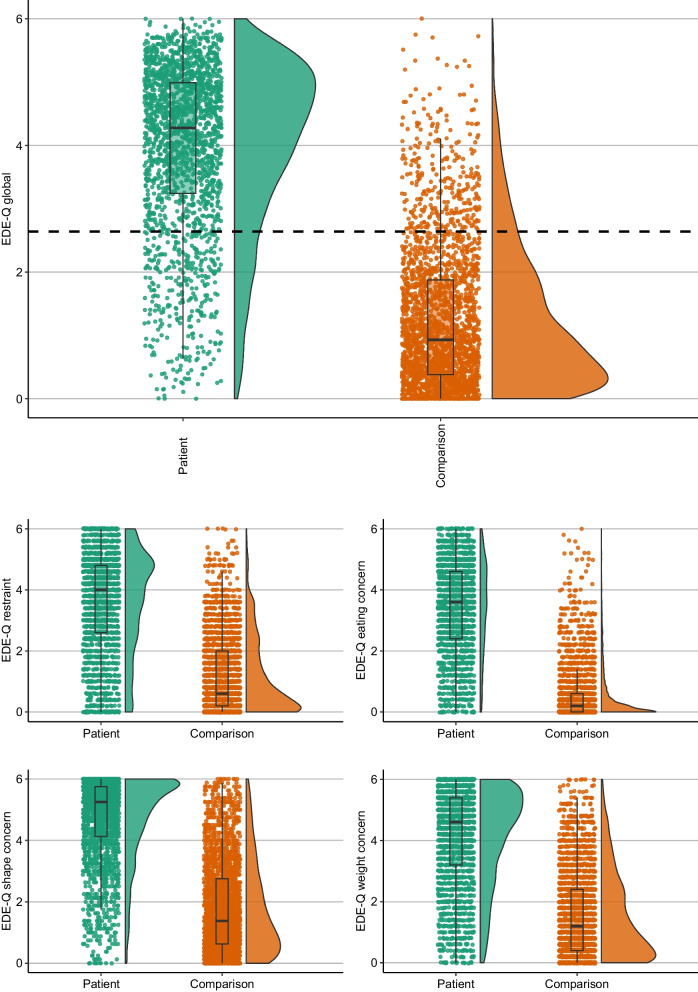
Raincloud plots showing distributions of EDE-Q global and subscale scores within patient and comparison groups. Dotted line signifies EDE-Q cut-off threshold (> 2.64)

Table 1 shows between-group differences in mean EDE-Q and EDE-Q7 global and subscale scores. The patient sample scored significantly higher on all global and subscale scores of the EDE-Q; and these differences were all of large magnitudes (*d*’s = 1.70–2.51). Similar results were observed for the EDE-Q7 global and subscale scores (with *d*’s = 1.38–1.88). Using non-parametric Mann–Whitney U tests did not change these results (see Additional file 1: Table S2). Both patients and comparisons scored higher on the EDE-Q7 compared to the EDE-Q. See Figs. 1 and 2 for graphs detailing the distributions of EDE-Q7 and EDE-Q global and subscale score across groups, and Additional file 1: Fig. S2 for a ridgeplot showing the distributions between groups across all individual EDE-Q items.

Visual inspection of the EDE-Q global and subscale score distributions illustrates that scores in both groups are heavily skewed in opposite directions. This applies to a lesser extent to the shape and weight concern subscales. EDE-Q7 distributions show similar patterns. However, the EDE-Q7 distributions among comparisons is less skewed than the EDE-Q. For example, scores on the EDE-Q7 subscale “body dissatisfaction” has a relatively non-skewed distribution among comparisons. Moreover, the EDE-Q7 global score has somewhat less extreme skewness compared to the EDE-Q.

### ROC analyses

The global EDE-Q score was able to discriminate between cases and comparisons with an AUC of 92% (95% CI 92–93%). The optimal combination of sensitivity and specificity was found at a global score of 2.64. A total of 84% of patients and 12% of comparisons scored above this threshold. The overall correct classification rate at this level was 86% (95% CI 85–87%), with a sensitivity of 84% and specificity of 88%. See Additional file 1: Fig. S3 for the ROC curve, and Additional file 1: Table S3 for ROC metrics for a range of EDE-Q global values.

**Fig. 3:**
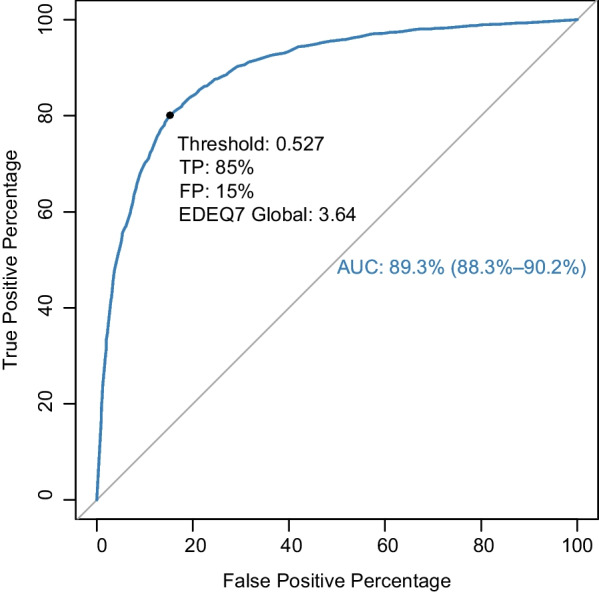
ROC curve showing the predictive ability of the EDE-Q7 to discriminate between patients and comparisons

The EDE-Q7 was similarly able to discriminate between patients and comparisons with an AUC of 89% (CI 88–90%). The optimal balance between sensitivity and specificity was achieved with a global score of 3.64. A total of 80% of patients and 15% of comparisons scored above this threshold. The overall correct classification rate at this level was 83% (95% CI 82–84%) with a sensitivity of 80% and specificity of 85%. See Fig. 3 for the ROC curve, and Additional file 1: Table S5 for ROC metrics for a range of EDE-Q7 global values. Exploratory ROC analyses (data not shown) of each diagnostic subgroup (i.e., AN, BN and other/unspecified ED) separately showed that the optimal cut-off threshold was identical for AN and BN (3.64) and slightly lower for other/unspecified ED (3.53).

### EDE-Q factor structure

The results of the confirmatory factor analysis for the full EDE-Q showed poor model fit for the four-factor solution for both patients (*χ*^2^ [202] = 3315, CFI = 0.877, TLI = 0.860, SRMR = 0.091, RMSEA = 0.092) and comparisons (*χ*^2^ [202] = 4412, CFI = 0.889, TLI = 0.873, SRMR = 0.097, RMSEA = 0.095); as well as for the total sample (*χ*^2^ [202] = 9038, CFI = 0.909, TLI = 0.895, SRMR = 0.066 & RMSEA = 0.103). The model fit for both patients and comparisons did not reach acceptable levels and was worse for the patient group.

### EDE-Q7 factor structure

The results of the confirmatory factor analysis showed satisfactory model for the three-factor solution for both patients (*χ*^2^ [10] = 50.57, CFI = 0.995, TLI = 0.990, SRMR = 0.023, RMSEA = 0.046) and comparisons (*χ*^2^ [10] = 35.85, CFI = 0.998, TLI = 0.996, SRMR = 0.013, RMSEA = 0.031). All fit indicators were within the normally suggested cut-offs [52]. The results from the factorial invariance test with exploratory factor analyses are presented in Additional file 1: Table S5. The results showed that factorial invariance could not be established as factor loadings for patients and comparisons differed notably. For patients the shape/weight overvaluation factor was not meaningfully different from the body dissatisfaction factor. As such, a two-factor solution was more parsimonious for patients, while a three factor was the optimal solution for comparisons. In addition, the multigroup configural invariance model showed a notable reduction in model fit (*χ*^*2*^ = 479.9 [31], CFI = 0.980, TLI = 0.973, SRMS = 0.092, RMSEA = 0.080), compared to the same model evaluated separately in the two groups. Because of this, we concluded that the instrument does not show configural invariance, and we did not proceed with tests of metric or scalar invariance.

The internal consistency was satisfactory for all three sub-scales in both groups (see Additional file 1: Table S6). In addition, the average variance extracted (AVE) showed satisfactory convergent validity for all three sub-scales across groups (see Additional file 1: Table S6). However, in the patient group, the squared correlation between body dissatisfaction and shape/weight overvaluation is higher than the AVE of both factors. As such, the high correlation violates the Fornell-Larcker criterion [53] and indicates a problem with discriminant validity between these two factors in patient populations.

## Discussion

We compared the psychometric properties of the brief EDE-Q7 with the full EDE-Q in a large sample of both patient and community comparisons and found that both the EDE-Q7 and full EDE-Q are adequately able to discriminate between clinical and non-clinical samples. Furthermore, we found support for the three-factor solution for the EDE-Q7 but failed to support the originally proposed four-factor solution of the full EDE-Q. Our findings show that the EDE-Q7 may be a viable alternative to the full EDE-Q and may be a particularly valuable assessment tool for non-clinical populations.

The EDE-Q7 produced slightly higher scores than the full EDE-Q in both patient and comparison groups. Both versions of the EDE-Q produced similar distributions of global and subscale scores, but there is a tendency for the EDE-Q7 to produce slightly less skewed scores among comparisons and more skewed scores among patients. This may be related to the fact that many patients and comparisons typically score relatively low on certain items (see Additional file 1: Fig. S2) which are omitted in the EDE-Q7. For example, this is the case for item 2 (i.e., how many days one has gone for long periods of time without eating anything at all) and item 19 (i.e., how many days one has eaten in secret). The fact that the EDE-Q7 produces higher scores may be beneficial for use in non-clinical populations, where it allows for more variation across participants. As such, the EDE-Q7 may be particularly suitable as an assessment of ED psychopathology in research settings comprising non-clinical samples. The EDE-Q7 may have less utility in clinical settings, where a detailed mapping of ED symptoms is desirable. Furthermore, for clinical work it may be worthwhile to include items covering height, weight, binge-eating and purging in addition to the seven items in the EDE-Q7. Further research is needed to examine whether the EDE-Q7 is sensitive to treatment effects and thus has utility as an outcome in treatment research. If a more thorough assessment of ED symptoms is desired within a brief format, the EDE-QS [16] may be a viable alternative.

Both the EDE-Q7 and full EDE-Q was able to reliably discriminate between patients and comparisons. The optimal global score cut-off threshold for discrimination was found to be 3.64 for the EDE-Q7. This is similar to the results obtained by Machado and colleagues [20] for the Portuguese version of the EDE-Q7, which showed an optimal cut-off threshold of 3.72. For the full EDE-Q, we found an optimal global score cut-off threshold of 2.64. The fact that the optimal cut-off threshold is one full point higher for the EDE-Q7 reflects that this version yields higher scores compared to the full EDE-Q. A total of 80% of patients and 15% of comparisons scored above the EDE-Q7 cut-off threshold, with an overall correct classification rate of 83%. This is slightly poorer compared to the full EDE-Q, where 84% of patients and 12% of comparisons scored above the cut-off threshold (with an overall correct classification rate of 86%). These findings support the use of the EDE-Q7 and full EDE-Q for crude discrimination purposes. However, the number of false positives and negatives is not negligible, which underscores the need to not overly rely on such cut-off thresholds. Although our approach using Youden’s statistic provides a quantitative index to determine a meaningful cut-off threshold, it is important to recognize that selection of an appropriate cut-off should not be solely dictated by this index. Other cut-off thresholds may be desirable in certain circumstances, for example if one wants to maximize either sensitivity or specificity. We have provided the full ROC tables in the Additional file 1, which may be of use to researchers who want to use a cut-off threshold to find the optimal balance between sensitivity and specificity for their needs. In the absence of any desire to optimize either sensitivity or specificity, the cut-off thresholds we propose may provide a balanced screening threshold. It should be noted that our results are based on analyses of clearly distinguished populations with diverging EDE-Q scores, where the patient sample represents the severe range of ED psychopathology. More classification errors would likely arise in settings where individuals are not as clearly demarcated (e.g., in epidemiological studies).

Our results supported the proposed three-factor solution of the EDE-Q7 but failed to support the originally proposed four-factor solution of the full EDE-Q. Failures to confirm the four-factor solution of the full EDE-Q are common [6]. Often the optimal solution is a variant of a three-factor solution [3, 7–15]. Indeed, such results prompted the development of the EDE-Q7 [10]. However, our factor analyses also showed that factorial invariance between groups could not be established for the EDE-Q7. For the patients a two-factor solution was more parsimonious, while a three-factor solution was optimal for comparisons. We therefore conclude that the EDE-Q7 does not show configural invariance. Care should therefore be exercised when comparing subscale scores between patient and comparison samples. This underscores the point that the EDE-Q7 may be more applicable in non-clinical contexts. Last, our findings supported the internal consistency of the three EDE-Q7 subscales.

While our study provides support for the psychometric properties and applicability of the EDE-Q7, it's important to note that our primary aim was not to compare the EDE-Q7 against other brief versions. Although the EDE-Q7 has undergone extensive testing, other brief versions such as the EDE-Q8 or EDE-QS may be viable alternatives. As previously mentioned, one limitation of the EDE-Q7 is its exclusion of all behavioral items from the original EDE-Q. In contexts where assessing behavioral features of eating disorders is crucial, the EDE-QS may prove especially valuable. It offers the additional advantage of focusing on the past seven days, which is particularly relevant for routine clinical assessments but may be of less importance in research settings. It's also worth mentioning that preliminary research has explored a version of the EDE-Q7 that includes the behavioral items from the EDE-Q [18, 36]. Furthermore, it's vital to acknowledge that self-report measures such as the EDE-Q7 are insufficient for establishing clinical diagnoses. Studies employing the EDE-Q7 as a general assessment tool for ED psychopathology should—if establishing case status is a priority—be followed by an appropriate diagnostic assessment.

Strengths of our study include the large sample size, inclusion of both clinical and non-clinical samples, and completeness of EDE-Q data. Our study also has several limitations. First, the patients in our sample represent the severe range of ED psychopathology, with 61% being in inpatient treatment and a high proportion being diagnosed with AN. This may have inflated the ability of the EDE-Q7 and EDE-Q in discriminating between our two groups. Also, our samples included few cases of binge-eating disorder. However, we note that previous studies have found that the EDE-Q7 performs well in this patient group as well [10, 25, 44]. Second, the community sample comprised samples from several studies, which differed in the recruitment and inclusion procedures. The extent to which these samples are representative of the community population is unclear. Although efforts were made to exclude ED cases from these samples it cannot be ruled out that some cases are included as comparisons. Moreover, our samples consisted entirely of females, and results are unlikely to generalize to male populations. Our sample is also unlikely to represent diversity in terms of gender identity and ethnicity. Third, the EDE-Q7 was not administered as a standalone measure but nested within the full EDE-Q. Administering only the 7 items in the EDE-Q7 may produce different scores compared to a nested version. Last, as cultural differences may impact EDE-Q scores it is important to establish norms and cut-off thresholds within a given cultural context.

## Conclusions

We showed that the brief EDE-Q7 performs close to the full EDE-Q in several respects. Although the EDE-Q7 produces higher scores than the full EDE-Q, distributions of scores are similar. Nevertheless, the EDE-Q7 was adequately able to discriminate between clinical and non-clinical samples. A cut-off threshold of 3.64 was established as optimal in discriminating between patients and comparisons. We also found support for the three-factor solution for the EDE-Q7, indicating good structural validity. Furthermore, the internal consistency of these three factors was satisfactory. Our findings indicate that the brief EDE-Q7 may be a viable alternative to the full EDE-Q in situations where response burden is an issue (e.g., epidemiological studies), and may be particularly suited for non-clinical populations. However, the EDE-Q7 may hold limited value over the full EDE-Q in clinical settings, due to the small number of items and lack of assessment of behavioral features.

### Supplementary Information

**Additional file 1: Table S1.** Number of valid observations and percentage missing for key variables. **Table S2.** Non-parametric test of between-group differences. **Table S3.** Specificity and sensitivity at various EDE-Q global cut-off thresholds. **Table S4.** Specificity and sensitivity at various EDE-Q7 global cut-off thresholds. **Figure S1.** Raincloud plots showing distributions of EDE-Q global scores and age across individual samples, colored according to group (patients vs. comparisons). Note: Samples 1-5 constitute samples from research studies, while studies 6-10 constitute samples from specialized eating disorder treatment units. EDE-Q = Eating Disorder Examination-Questionnaire. **Figure S2.** Ridgeplot showing distributions of scores on individual items across groups. *Note*: The EDE-Q7 comprise items 1, 3, 4 (dietary restraint), 22, 23 (shape/weight overvaluation), 25 and 26 (body dissatisfaction). EDE-Q = Eating Disorder Examination-Questionnaire. **Figure S3.** ROC curve showing the predictive ability of the EDE-Q to discriminate between patients and comparisons. *Note*: EDE-Q = Eating Disorder Examination-Questionnaire, TP = true positive, FP = false positive, AUC = area under curve. **Table S5.** Confirmatory factor analysis with three factors assessing configural invariance of EDE-Q 7 across patient and comparison samples. **Table S6.** Internal consistency, Average Variance Extracted (AVE) and inter-correlations between factors.

## Data Availability

The datasets used and/or analyzed during the current study are available from the corresponding author on reasonable request.

## Abbreviations

AN: Anorexia nervosa
AUC: Area under curve
AVE: Average variance extracted
BMI: Body mass index
BN: Bulimia nervosa
CFI: Comparative fit index
CI: Confidence interval
ED: Eating disorder
EDE-Q: Eating Disorder Examination-Questionnaire
ICD: International classification of diseases
RMSEA: Root mean square error of approximation
ROC: Receiver operator curve
SRMR: Standardized root mean squared residual
TLI: Tucker–Lewis index
