# Supplementary material for: Comparison between the brief seven-item and full eating disorder examination-questionnaire (EDE-Q) in clinical and non-clinical female Norwegian samples
Source: J Eat Disord. 2023 Nov 2;11:194. doi: 10.1186/s40337-023-00920-x (PMC10621120; doi:10.1186/s40337-023-00920-x)
Supplement: Supplementary file 1 — Additional file 1: Table S1. Number of valid observations and percentage missing for key variables. Table S2. Non-parametric test of between-group differences. Table S3. Specificity and sensitivity at various EDE-Q global cut-off thresholds. Table S4. Specificity and sensitivity at various EDE-Q7 global cut-off thresholds. Figure S1. Raincloud plots showing distributions of EDE-Q global scores and age across individual samples, colored according to group (patients vs. comparisons). Note: Samples 1-5 constitute samples from research studies, while studies 6-10 constitute samples from specialized eating disorder treatment units. EDE-Q = Eating Disorder Examination-Questionnaire. Figure S2. Ridgeplot showing distributions of scores on individual items across groups. Note: The EDE-Q7 comprise items 1, 3, 4 (dietary restraint), 22, 23 (shape/weight overvaluation), 25 and 26 (body dissatisfaction). EDE-Q = Eating Disorder Examination-Questionnaire. Figure S3. ROC curve showing the predictive ability of the EDE-Q to discriminate between patients and comparisons. Note: EDE-Q = Eating Disorder Examination-Questionnaire, TP = true positive, FP = false positive, AUC = area under curve. Table S5. Confirmatory factor analysis with three factors assessing configural invariance of EDE-Q 7 across patient and comparison samples. Table S6. Internal consistency, Average Variance Extracted (AVE) and inter-correlations between factors. [file 40337_2023_920_MOESM1_ESM.docx]

**Table S1. Number of valid observations and percentage missing for key variables.**

|  | Patient (*n* = 1954) | Comparison (*n* = 2430) |
| --- | --- | --- |
| Variable | *Valid n* (% missing) | *Valid n* (% missing) |
| Age | 1612 (17.5%) | 2403 (1.1%) |
| BMI (kg/m^2^) | 1688 (13.6%) | 2387 (1.8%) |
| EDE-Q global | 1952 (0.1%) | 2430 (0.0%) |
| EDE-Q restraint | 1954 (0.0%) | 2429 (0.0%) |
| EDE-Q eating concern | 1945 (0.5%) | 2429 (0.1%) |
| EDE-Q weight concern | 1948 (0.3%) | 2430 (0.0%) |
| EDE-Q shape concern | 1948 (0.3%) | 2429 (0.0%) |
| EDE-Q7 global | 1900 (2.8%) | 2370 (2.5%) |
| EDE-Q7 dietary restraint | 1937 (0.9%) | 2407 (0.9%) |
| EDE-Q7 shape/weight overvaluation | 1933 (1.1%) | 2418 (0.5%) |
| EDE-Q7 body dissatisfaction | 1926 (1.4%) | 2404 (1.1%) |

Note: BMI = body mass index, EDE-Q = Eating Disorder Examination-Questionnaire.

**Table S2. Non-parametric test of between-group differences.**

|  | Sample descriptives | | Mann-Whitney U | |
| --- | --- | --- | --- | --- |
| Variable | **Patient**  **(*n* = 1954)** | **Comparison**  **(*n* = 2430)** | ***W*** | ***p*** |
|  | ***M_dn_* (IQR)** | ***M_dn_* (IQR)** |  |  |
| Age | 26.00 (11.90) | 30.00 (18.00) | 1605241 | <.001 |
| BMI | 19.80 (6.15) | 22.80 (4.90) | 1186247 | <.001 |
| EDE-Q global | 4.28 (1.75) | 0.93 (1.50) | 4375881 | <.001 |
| EDE-Q restraint | 4.00 (2.20) | 0.60 (1.80) | 4093434 | <.001 |
| EDE-Q eating concern | 3.60 (2.20) | 0.20 (0.60) | 4466971 | <.001 |
| EDE-Q weight concern | 4.60 (2.20) | 1.20 (2.00) | 4149878 | <.001 |
| EDE-Q shape concern | 5.25 (1.62) | 1.38 (2.12) | 4267782 | <.001 |
| EDE-Q7 global | 5.00 (1.67) | 1.56 (2.22) | 4019920 | <.001 |
| EDE-Q7 dietary restraint | 5.00 (3.00) | 1.00 (2.67) | 3818711 | <.001 |
| EDE-Q7 shape/weight overvaluation | 5.00 (2.00) | 1.50 (2.50) | 4131153 | <.001 |
| EDE-Q7 body dissatisfaction | 5.50 (2.00) | 2.00 (2.50) | 3880356 | <.001 |

Note: Results considered statistically significant if *p* < .05. BMI = body mass index, EDE-Q = Eating Disorder Examination-Questionnaire, IQR = inter-quartile range, M_dn_ = median.

**Table S3. Specificity and sensitivity at various EDE-Q global cut-off thresholds.**

| EDE-Q global | Threshold | Specificity | Sensitivity | Accuracy |
| --- | --- | --- | --- | --- |
| 1.0 | 9.2 | 53.0 | 96.3 | 72.3 |
| 1.1 | 10.4 | 56.3 | 96.1 | 74.0 |
| 1.2 | 11.7 | 59.7 | 95.6 | 75.7 |
| 1.3 | 13.1 | 61.9 | 95.1 | 76.7 |
| 1.4 | 14.7 | 63.8 | 94.2 | 77.3 |
| 1.5 | 16.5 | 66.4 | 93.6 | 78.5 |
| 1.6 | 18.4 | 69.1 | 92.9 | 79.7 |
| 1.7 | 20.5 | 71.6 | 92.3 | 80.8 |
| 1.8 | 22.8 | 73.7 | 91.6 | 81.7 |
| 1.9 | 25.2 | 75.8 | 91.0 | 82.6 |
| 2.0 | 27.8 | 77.9 | 90.2 | 83.4 |
| 2.1 | 30.6 | 79.8 | 88.7 | 83.8 |
| 2.2 | 33.5 | 81.7 | 87.5 | 84.3 |
| 2.3 | 36.5 | 82.8 | 86.7 | 84.6 |
| 2.4 | 39.7 | 84.7 | 85.5 | 85.1 |
| 2.5 | 42.9 | 85.7 | 85.0 | 85.4 |
| 2.6 | 46.2 | 87.0 | 84.1 | 85.7 |
| 2.7 | 49.5 | 87.9 | 82.8 | 85.6 |
| 2.8 | 52.9 | 89.3 | 81.5 | 85.9 |
| 2.9 | 56.2 | 90.1 | 79.9 | 85.5 |
| 3.0 | 59.4 | 91.4 | 79.0 | 85.9 |
| 3.1 | 62.6 | 92.0 | 77.6 | 85.6 |
| 3.2 | 65.7 | 92.7 | 75.6 | 85.1 |
| 3.3 | 68.6 | 93.5 | 74.0 | 84.8 |
| 3.4 | 71.4 | 94.2 | 72.7 | 84.6 |
| 3.5 | 74.1 | 94.8 | 70.4 | 83.9 |
| 3.6 | 76.6 | 95.5 | 68.3 | 83.4 |
| 3.7 | 78.9 | 95.9 | 66.3 | 82.7 |
| 3.8 | 81.0 | 96.4 | 63.3 | 81.7 |
| 3.9 | 83.0 | 96.8 | 60.6 | 80.7 |
| 4.0 | 84.8 | 97.2 | 58.1 | 79.8 |
| 4.1 | 86.4 | 97.6 | 55.2 | 78.7 |
| 4.2 | 87.9 | 97.9 | 52.6 | 77.7 |
| 4.3 | 89.3 | 98.1 | 48.9 | 76.2 |
| 4.4 | 90.5 | 98.6 | 46.1 | 75.2 |
| 4.5 | 91.6 | 98.8 | 43.1 | 74.0 |
| 4.6 | 92.6 | 99.1 | 38.9 | 72.3 |
| 4.7 | 93.4 | 99.1 | 35.5 | 70.8 |
| 4.8 | 94.2 | 99.3 | 32.2 | 69.4 |
| 4.9 | 94.9 | 99.5 | 28.7 | 68.0 |
| 5.0 | 95.5 | 99.5 | 24.9 | 66.3 |
| 5.1 | 96.0 | 99.5 | 21.3 | 64.7 |
| 5.2 | 96.5 | 99.5 | 16.7 | 62.6 |
| 5.3 | 96.9 | 99.7 | 14.4 | 61.7 |
| 5.4 | 97.3 | 99.8 | 10.2 | 59.9 |
| 5.5 | 97.6 | 99.8 | 7.2 | 58.6 |
| 5.6 | 97.9 | 99.8 | 4.8 | 57.5 |
| 5.7 | 98.2 | 99.9 | 1.7 | 56.2 |
| 5.8 | 98.4 | 100.0 | 1.0 | 55.9 |
| 5.9 | 98.6 | 100.0 | 0.6 | 55.7 |
| 6.0 | 98.8 | 100.0 | 0.0 | 55.5 |

Note: EDE-Q = Eating Disorder Examination-Questionnaire.

**Table S4. Specificity and sensitivity at various EDE-Q7 global cut-off thresholds.**

| EDEQ7 Global | Threshold | Specificity | Sensitivity | Accuracy |
| --- | --- | --- | --- | --- |
| 1.0 | 6.9 | 36.5 | 97.5 | 63.7 |
| 1.1 | 7.6 | 37.6 | 97.4 | 64.2 |
| 1.2 | 8.3 | 40.2 | 97.2 | 65.5 |
| 1.3 | 9.1 | 42.9 | 97.0 | 67.0 |
| 1.4 | 10.0 | 46.5 | 96.3 | 68.6 |
| 1.5 | 11.0 | 49.3 | 95.8 | 70.0 |
| 1.6 | 12.0 | 50.4 | 95.6 | 70.5 |
| 1.7 | 13.2 | 53.5 | 95.2 | 72.0 |
| 1.8 | 14.4 | 55.4 | 94.7 | 72.9 |
| 1.9 | 15.7 | 58.0 | 94.4 | 74.2 |
| 2.0 | 17.1 | 60.2 | 93.3 | 74.9 |
| 2.1 | 18.6 | 61.0 | 93.1 | 75.2 |
| 2.2 | 20.2 | 63.2 | 92.7 | 76.3 |
| 2.3 | 21.9 | 64.6 | 92.3 | 76.9 |
| 2.4 | 23.7 | 67.3 | 91.5 | 78.0 |
| 2.5 | 25.6 | 68.4 | 91.2 | 78.5 |
| 2.6 | 27.6 | 70.1 | 90.4 | 79.2 |
| 2.7 | 29.7 | 72.4 | 89.1 | 79.8 |
| 2.8 | 31.9 | 74.0 | 88.2 | 80.3 |
| 2.9 | 34.2 | 75.5 | 87.6 | 80.9 |
| 3.0 | 36.6 | 77.1 | 86.2 | 81.1 |
| 3.1 | 39.0 | 77.6 | 86.1 | 81.4 |
| 3.2 | 41.4 | 79.4 | 84.5 | 81.7 |
| 3.3 | 44.0 | 80.7 | 83.7 | 82.1 |
| 3.4 | 46.5 | 82.4 | 81.9 | 82.2 |
| 3.5 | 49.1 | 83.8 | 80.9 | 82.5 |
| 3.6 | 51.6 | 84.3 | 80.4 | 82.6 |
| 3.7 | 54.2 | 85.8 | 78.6 | 82.6 |
| 3.8 | 56.7 | 86.7 | 77.1 | 82.4 |
| 3.9 | 59.2 | 87.4 | 75.8 | 82.2 |
| 4.0 | 61.7 | 88.6 | 72.8 | 81.5 |
| 4.1 | 64.1 | 88.9 | 72.3 | 81.5 |
| 4.2 | 66.4 | 90.0 | 70.1 | 81.1 |
| 4.3 | 68.7 | 91.0 | 68.0 | 80.7 |
| 4.4 | 70.8 | 91.8 | 64.9 | 79.8 |
| 4.5 | 72.9 | 92.2 | 63.6 | 79.5 |
| 4.6 | 74.9 | 93.0 | 60.2 | 78.4 |
| 4.7 | 76.8 | 93.9 | 57.0 | 77.5 |
| 4.8 | 78.6 | 94.6 | 55.6 | 77.3 |
| 4.9 | 80.2 | 95.3 | 52.0 | 76.0 |
| 5.0 | 81.8 | 95.5 | 51.5 | 75.9 |
| 5.1 | 83.3 | 96.4 | 47.5 | 74.6 |
| 5.2 | 84.7 | 96.8 | 43.9 | 73.3 |
| 5.3 | 86.0 | 97.0 | 41.7 | 72.4 |
| 5.4 | 87.2 | 97.8 | 34.9 | 69.8 |
| 5.5 | 88.3 | 98.0 | 31.6 | 68.5 |
| 5.6 | 89.3 | 98.3 | 29.2 | 67.5 |
| 5.7 | 90.2 | 98.7 | 24.1 | 65.5 |
| 5.8 | 91.1 | 98.9 | 21.6 | 64.5 |
| 5.9 | 91.9 | 99.1 | 16.6 | 62.4 |
| 6.0 | 92.6 | 99.1 | 16.6 | 62.4 |

Note: EDE-Q = Eating Disorder Examination-Questionnaire.


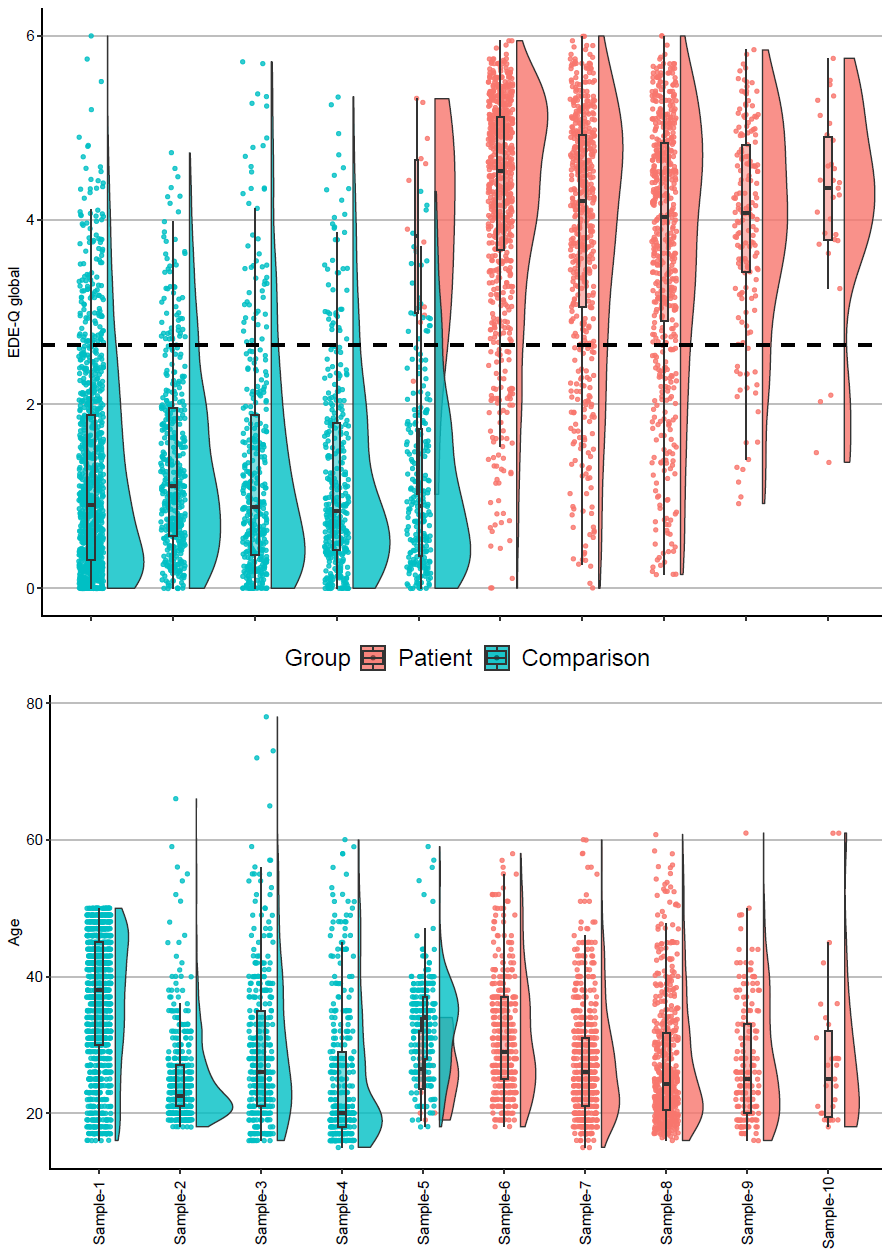


**Figure S1. Raincloud plots showing distributions of EDE-Q global scores and age across individual samples, colored according to group (patients vs. comparisons).** Note: Samples 1-5 constitute samples from research studies, while studies 6-10 constitute samples from specialized eating disorder treatment units. EDE-Q = Eating Disorder Examination-Questionnaire.

**
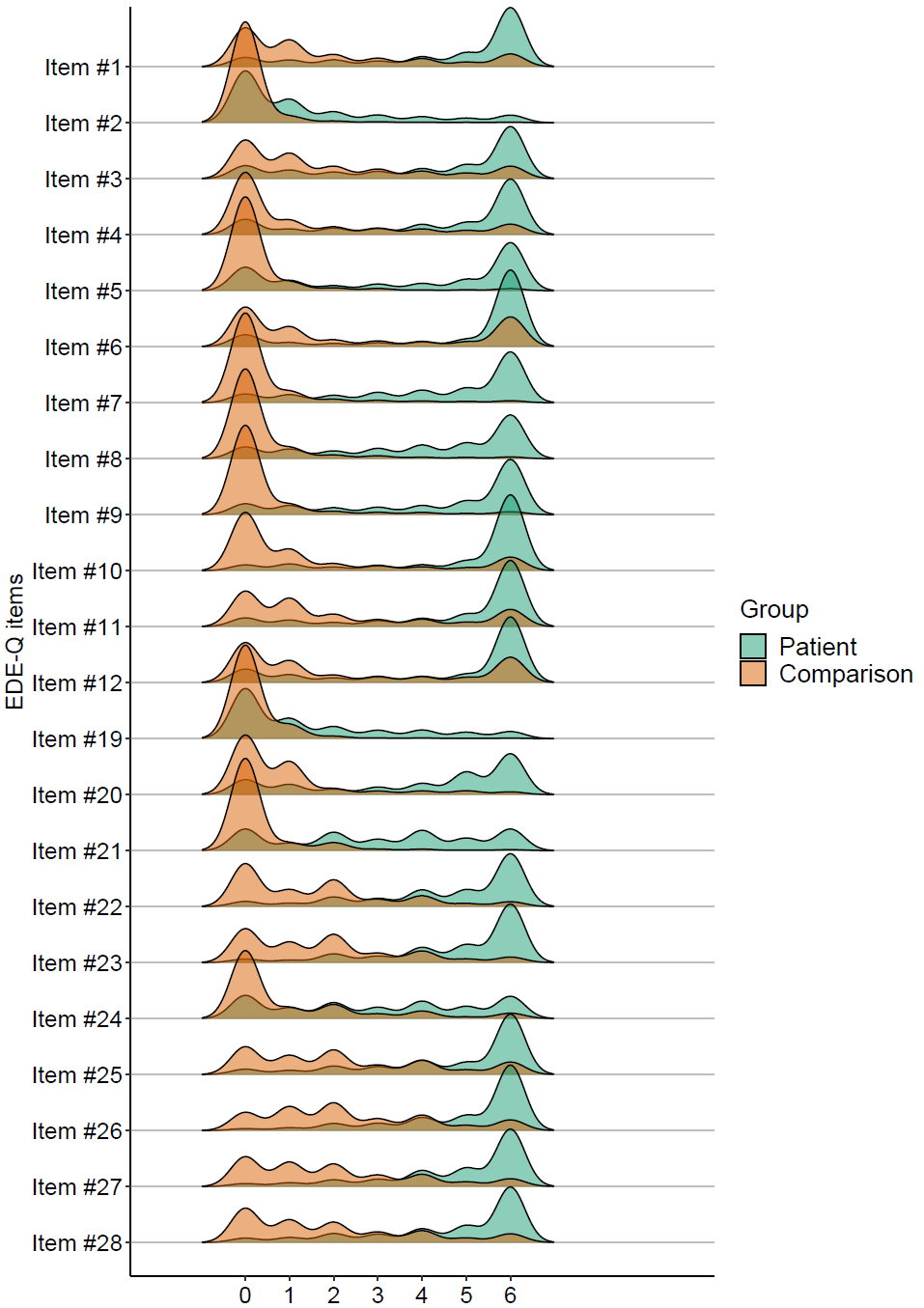
**

**Figure S2. Ridgeplot showing distributions of scores on individual items across groups.** Note: The EDE-Q7 comprise items 1, 3, 4 (dietary restraint), 22, 23 (shape/weight overvaluation), 25 and 26 (body dissatisfaction). EDE-Q = Eating Disorder Examination-Questionnaire.


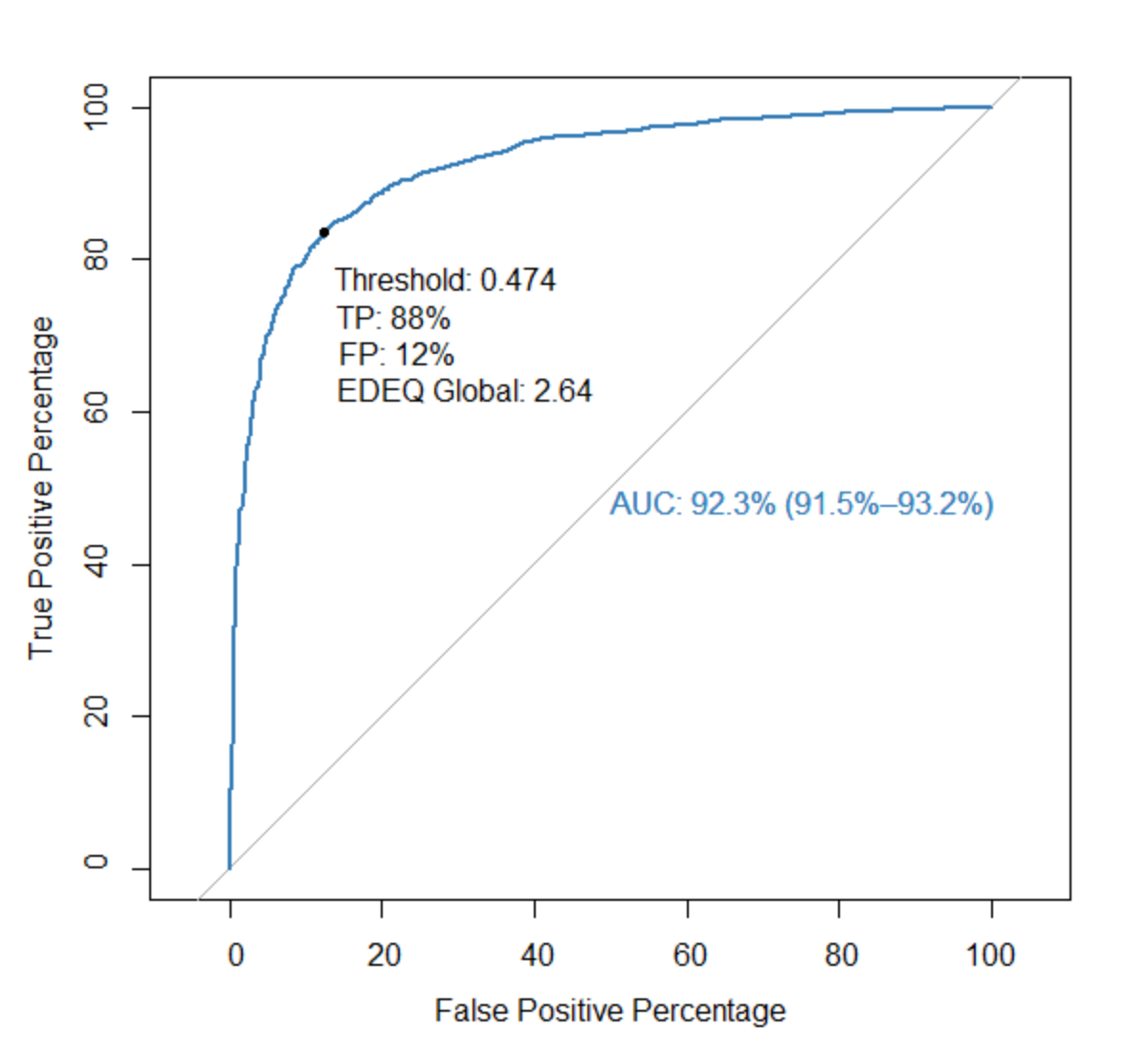


**Figure S3. ROC curve showing the predictive ability of the EDE-Q to discriminate between patients and comparisons.** Note: EDE-Q = Eating Disorder Examination-Questionnaire, TP = true positive, FP = false positive, AUC = area under curve.

| **Table S5. Confirmatory factor analysis with three factors assessing configural invariance of EDE-Q 7 across patient and comparison samples.** | | | | | | |
| --- | --- | --- | --- | --- | --- | --- |
| Item | Factor 1 (dietary restraint) | | Factor 2 (shape/weight overvaluation) | | Factor 3 (body dissatisfaction) | |
|  | Patients | Comparisons | Patients | Comparisons | Patients | Comparisons |
| EDE-Q #1 | **.77** | **.75** | -.01 | .03 | .05 | .09 |
| EDE-Q #3 | **.80** | **.78** | .01 | .02 | .04 | .01 |
| EDE-Q #4 | **.84** | **.77** | -.01 | .02 | -.04 | -.01 |
| EDE-Q #22 | .02 | .03 | .36 | **.78** | **.70** | .15 |
| EDE-Q #23 | .02 | .03 | .38 | **.84** | **.67** | .06 |
| EDE-Q #25 | .01 | .05 | -.05 | .09 | **.84** | **.80** |
| EDE-Q #26 | .00 | .02 | -.03 | .17 | **.84** | **.76** |
| Note: Values in bold denote the highest loading of each variable within the patient and comparison groups. EDE-Q = Eating Disorder Examination-Questionnaire. | | | | | | |

| **Table S6. Internal consistency, Average Variance Extracted (AVE) and inter-correlations between factors.** | | | | | | | | | | |
| --- | --- | --- | --- | --- | --- | --- | --- | --- | --- | --- |
| Factor | Comparisons | | | | | Patients | | | | |
|  | *ρ* | AVE | 1 | 2 | 3 | *ρ* | AVE | 1 | 2 | 3 |
| 1: Dietary restraint | .86 | .671 | - | - | - | .87 | .693 | - | - | - |
| 2: Shape/weight overvaluation | .97 | .855 | .59 | - | - | .97 | .795 | .44 | - | - |
| 3: Body dissatisfaction | .92 | .853 | .61 | .82 | - | .86 | .749 | .36 | .88 | - |
| Note: All correlations are significant at p < .01. *ρ* = Raykov's reliability coefficient. | | | | | | | | | | |
